# Buttressing the Staple Line: A Randomized Comparison Between Staple-Line Reinforcement Versus No Reinforcement During Sleeve Gastrectomy

**DOI:** 10.1007/s11695-014-1374-z

**Published:** 2014-08-17

**Authors:** Shashank S. Shah, Jayashree S. Todkar, Poonam S. Shah

**Affiliations:** 1 Department of Laparoscopic and Bariatric Surgery, Dr. L. H. Hiranandani Hospital, Mumbai, India; 2 Ruby Hall Clinic, Laparo-Obeso Centre, 40, Sassoon Road, Pune, 411001 India; 3 Poona Hospital, 27, Sadashiv Peth, Pune, 411001 India

**Keywords:** Sleeve gastrectomy, Staple line, Buttress, Bariatric surgery, Peri-Strips, PSD-V, Peri-Strips Dry® with Veritas®

## Abstract

Bariatric surgery is recommended for Indian patients with body mass index (BMI) >32.5 kg/m^2^ with at least one comorbidity and >37.5 kg/m^2^ without a comorbidity. In laparoscopic sleeve gastrectomy, bleeding and leakage from the staple line are common post-operative events. Peri-Strips Dry® with Veritas® (PSD-V) is used in staple-line reinforcement. This was a single-investigator, multicenter, randomized study of 100 patients undergoing standard sleeve gastrectomy with a 34 or 36 French bougie. Patients were randomized 1:1 to PSD-V or control groups; no buttress material was used in the control group. The primary objective was to assess complication rates (any staple-line bleed or leak from the intra-operative visit through day 30) associated with sleeve gastrectomy. Surgical time (from first incision to closure of last incision) and the number of clips and/or sutures used to control bleeding were also assessed. Fewer staple-line bleeds were observed in the PSD-V group than the control group (23/51 [45.1 %] vs 39/49 [79.6 %] patients; *p* = 0.0005), and the bleeding was of a lower severity (*p* = 0.0002). No staple-line leaks were observed. Surgical time was shorter in patients who received PSD-V (58.8 vs 72.8 min; *p* = 0.0153), and fewer patients required hemostatic clips and/or sutures (10/51 [19.6 %] vs 33/49 [67.3 %] patients; *p* < 0.0001). Fewer patients in the PSD-V than the control group experienced adverse events (2/51 [3.9 %] vs 5/49 [10.2 %] patients). The use of PSD-V reduced the incidence and severity of staple-line bleeding and was associated with a reduction in surgical time compared with no staple-line reinforcement.

## Introduction

The World Health Organization reports that >1.4 billion adults are overweight and that nearly 500 million are obese,[1] contributing to a global increase in obesity-related comorbidities, including hypertension, type 2 diabetes mellitus, and cardiovascular disease [2].

Obesity often occurs in developing countries in tandem with under-nutrition, affecting nearly all ages and socioeconomic groups [1]. In 2005–2006, 12.6 % of women and 9.7 % of men were considered overweight or obese in India, based on a body mass index (BMI) ≥25.0 [3]. However, it is now recognized that Asian Indians are at greater risk of obesity-related comorbidities at lower levels of BMI and waist circumference/waist-to-hip ratio; therefore, alternative cutoff points for definitions of “overweight” and “obese” may be more appropriate [4, 5]. It is now recommended that an Indian is considered overweight with a BMI of 23.0–24.9 kg/m^2^ and obese with a BMI >25 kg/m^2^ [6].

The Consensus Statement for Asian Indians recommends bariatric surgery for patients with a BMI >32.5 kg/m^2^ and a comorbidity and for patients who have a BMI >37.5 kg/m^2^ without a comorbidity [6]. Sleeve gastrectomy evolved from a larger gastric component of the duodenal switch with biliopancreatic diversion and is now widely accepted as a primary bariatric operation [7]. The procedure has yielded promising mid-term data, with reductions in BMI at 6 and 12 months post-surgery, in conjunction with cure of or improvement in major comorbidities [8].

Bleeding and leakage are common complications following sleeve gastrectomy due to the long staple line. A variety of intra-operative methods are used in preventing these events, including over-sewing of staple lines and use of linear staplers with shorter staple height. Staple-line reinforcement is also beneficial, with reductions in operative time, limitation of post-operative gastric leakage and bleeding, and fewer post-operative complications [9, 10], though some surgeons also use sutures or clips for control of hemorrhage.

A number of materials have been studied in staple-line reinforcement, including porcine small intestinal submucosa strips [11], absorbable polyglycolic acid [12], and bovine pericardial strips [13, 14]. The use of integrated absorbable synthetic polymers has proved feasible and is well tolerated [15], and glycolide copolymer reinforcement sleeves have been shown to reduce staple-line bleeding and may reduce gastrointestinal hemorrhaging [16]. Dapri et al. reported less blood loss with the use of glycolide trimethylene carbonate copolymer during stomach sectioning, although a recent study using the same material showed no significant difference in bleeding and post-operative leaks versus staple-line suturing [17, 18].

Peri-Strips Dry® with Veritas® (PSD-V; Synovis Life Technologies, St. Paul, MN, USA) is an implantable biologic tissue composed of non-cross-linked bovine pericardium, treated with sodium hydroxide to reduce or inactivate transmissible spongiform encephalopathies/bovine spongiform encephalopathies. The use of bovine pericardium strips has been shown to reduce staple-line bleeding, with fewer days of hospitalization compared with patients with no staple-line reinforcement [14]. Recently, Hajj et al. 2013 reported that bovine pericardium strips were associated with a lower staple-line leak rate compared with over-sewing [19]. PSD-V, approved in the USA, has received the CE Mark (indicating product compliance with European Union legislation) and has recently been approved in India.

This clinical trial evaluated the benefits of using PSD-V as a staple-line reinforcement during sleeve gastrectomy surgery and in preventing staple-line bleeds and leaks within the first 30 post-operative days.

## Materials and Methods

### Study Design

This was a single-investigator, multicenter, randomized study conducted at two sites (Ruby Hall Clinic and Poona Hospital and Research Centre, Pune, India), with surgeries performed between June 2011 and April 2012. The same surgical team was present at both study sites.

Study methods were in compliance with the Indian Council of Medical Research regulations (Ethical Guidelines for Biomedical Research on Human Participants) [20] and the Declaration of Helsinki [21]. The study was approved by the Ethics Committee of Poona Hospital and Research Centre and by the Ethics Committee of Poona Medical Research and Foundation. Study-specific informed consent forms were provided for patients in three languages: English, Hindi, and Marathi.

### Patients

Inclusion and exclusion criteria for the study were in accordance with guidelines for bariatric surgery [7]. Key inclusion criteria included patient age between 18 and 75 years inclusive, a BMI >32.5 kg/m^2^ with one or more comorbidities, or a BMI >37.5 kg/m^2^ with no comorbidities. In addition, the primary investigator had to consider the patient a good candidate for sleeve gastrectomy surgery. Exclusion criteria included uncontrolled hypertension, current drug or alcohol abuse, uncontrolled severe psychiatric illness, and disorders that may contribute to obesity.

### Procedures

Patients were randomized to PSD-V or control groups in a 1:1 ratio, with randomization performed using sealed envelopes that were opened immediately prior to surgery. The randomization block size was 6 and no randomization errors occurred.

Standard sleeve gastrectomy was performed using 34 or 36 French (Fr) bougies, consistent with guidelines reporting an optimal bougie size of 32–36 Fr bougies [22]; the choice of a 34 or 36 Fr bougie was that of the operating surgeon and was not pre-specified in the protocol. An Ethicon Echelon Linear Surgical Stapler (Ethicon Endo-Surgery, Cincinnati, OH, USA) was used in all cases, with 30 s of compression before firing to exude fluid from the tissue and thereby ensure a more uniform tissue thickness. In the group that received staple-line reinforcement with PSD-V, only green staple cartridges (2.0 mm) were used. In the control group, no buttress material was used; the staples in the lower part of the stomach were green (2.0 mm); and the staples in the upper part of the stomach were blue (1.5 mm), consistent with findings that tissue is thickest at the antrum and thinner toward the fundus [23]. Green staples are more appropriate for thicker tissue, as their wider diameter ensures they are stronger, and they form a longer leg length [24]. In the absence of buttress material, sutures or clips were used at the point of bleeding. Drains were routinely placed in any patient considered to be at higher medical risk of complications, as a safety measure. Patients underwent post-operative assessment on days 1, 7, and 30.

### Objectives

The primary objective was to evaluate the complication rates associated with sleeve gastrectomy, defined as any reported staple-line bleed or leak from an intra-operative visit through day 30. Intra-operative leaks were assessed using a methylene blue and/or an air-leak test.

The following severity scale was used for the grading of intra-operative staple-line bleeds:

- No bleed—the patient did not require a transfusion or intervention for bleeding during the operation
- Mild bleed—a change in laboratory values that resolved without further intervention
- Moderate bleed—a change in laboratory values with bleeding that required clips, sutures, or stitches, or that required a blood transfusion
- Severe bleed—the patient required an intervention, such as a return to the operating room to correct a staple-line bleed

The decision to transfuse was based on a combination of clinical evaluation, changes in hemoglobin levels, and hemodynamic monitoring. Bleeding severity was also assessed by recording the number of bleeding points over the staple line; in each case, the number of clips or sutures used to achieve hemostasis for these bleeding points was recorded, as the number of bleeding points was not necessarily the same as the number of clips/sutures.

The secondary objective was to prospectively evaluate the surgical time (from the first incision to the closure of the last incision) and the number of clips and/or sutures used to control bleeding in the PSD-V group versus the control group. The time required for induction and reversal of anesthesia was excluded to demonstrate real surgical time differences. Adverse events (AEs) were assessed throughout the study period.

### Data Analysis

The sample size was not statistically driven, as >1,000 patients would be needed to reach statistical significance for post-operative bleeds and gastric leaks. Data were summarized by study arm using descriptive statistics (mean, standard deviation [SD], median, interquartile range, range, counts, and percentages), and 95 % confidence intervals were calculated for the primary study objective. Fisher’s exact test was used to compare categorical measures between the study arms, while for continuous measures, comparisons were performed using Wilcoxon rank-sum tests.

The severity of the patients’ worst bleed was compared between groups using the Wilcoxon rank-sum test; patients with no bleeds were given a score of 0, and patients with a worst bleed severity of mild, moderate, or severe were assigned scores of 1, 2, or 3, respectively. Nominal *p* values were reported, and no adjustments for multiple comparisons were made.

## Results

Of the 100 patients who consented to study participation and who were randomized to treatment, 46 were male and 54 were female. All patients were followed to the end of the 30-day study period. Patient baseline characteristics (Table 1) and comorbidities (Table 2) were generally well balanced between the treatment arms, but there were more males in the PSD-V group than the control group (*p* = 0.0099). PSD-V was used along the length of the staple line in each sleeve gastrectomy; the mean (±SD) number of PSD-V strips used in each case was 6.9 ± 1.2.

**Table 1:** Patient baseline characteristics

		PSD-V, *N* = 51	Control, *N* = 49	*p* value^a^
Age (years)	Mean ± SD	39.2 ± 14.6	36.0 ± 11.6	0.2087
	Median (IQR)	38.2 (27.5, 50.0)	33.6 (26.9, 46.3)	
Male gender	% (n/N)	58.8 (30/51)	32.7 (16/49)	0.0099
Race				
Asian Indian	% (n/N)	98.0 (50/51)	100.0 (49/49)	1.0000
African	% (n/N)	2.0 (1/51)	0.0 (0/49)	1.0000
Height (cm)	Mean ± SD	164.5 ± 8.6	163.3 ± 8.7	0.5047
	Median (IQR)	163.0 (157.0, 172.4)	164.0 (157.0, 168.0)	
	Range	151.0–185.0	143.0–186.0	
Weight (kg)	Mean ± SD	124.9 ± 24.9	119.4 ± 29.8	0.1699
	Median (IQR)	122.8 (105.0, 144.0)	110.0 (100.8, 136.0)	
	Range	83.0–179.3	74.0–219.0	
BMI	Mean ± SD	46.1 ± 8.5	44.7 ± 9.8	0.1967
	Median (IQR)	43.6 (40.1, 51.1)	40.9 (38.0, 51.4)	
	Range	34.4–69.2	32.9–75.8	

^a^Wilcoxon rank-sum test used to compare continuous measures between groups; Fisher’s exact test used to compare categorical measures between groups

*BMI* body mass index, *IQR* interquartile range, *PSD*-*V* Peri-Strips Dry® with Veritas®, *SD* standard deviation

**Table 2:** Patient comorbidities

	PSD-V, *N* = 51	Control, *N* = 49	*p* value^a^
Medical comorbidities, % (n/N)			
Hypertension	29.4 (15/51)	36.7 (18/49)	0.5248
Hyperlipidemia	3.9 (2/51)	8.2 (4/49)	0.4316
Diabetes mellitus	23.5 (12/51)	20.4 (10/49)	0.8107
Sleep disorders	58.8 (30/51)	53.1 (26/49)	0.6873
GERD	0.0 (0/51)	2.0 (1/49)	0.4900
Depression	11.8 (6/51)	8.2 (4/49)	0.7412
Previous gastric surgery	0.0 (0/51)	2.0 (1/49)	0.4900
Other diseases and/or surgery, % (n/N)			
Renal	0.0 (0/51)	4.1 (2/49)	0.2376
Gastrointestinal	2.0 (1/51)	4.1 (2/49)	0.6136
Respiratory	70.6 (36/51)	67.3 (33/49)	0.8296
Musculoskeletal	41.2 (21/51)	34.7 (17/49)	0.5417
Cardiovascular	11.8 (6/51)	24.5 (12/49)	0.1218
Other	94.1 (48/51)	95.9 (47/49)	1.0000

*GERD* gastroesophageal reflux disease, *PSD*-*V* Peri-Strips Dry® with Veritas®

^a^Fisher’s exact test used to compare groups

No intra-operative or post-operative leaks were reported in either treatment arm. Fewer staple-line bleeds were observed in the PSD-V group (23/51 [45.1 %] vs 39/49 [79.6 %] patients), and this difference reached statistical significance (*p* = 0.0005; Table 3). Subgroup analyses in patients with a BMI <43 also demonstrated fewer bleeds in the PSD-V group compared with the control group (7/21 [33.3 %] vs 23/29 [79.3 %] patients; *p* = 0.0015; Table 4). However, in patients with a BMI ≥43, there was no significant difference between the PSD-V and control groups (16/30 [53.3 %] vs 16/20 [80.0 %] patients; *p* = 0.0742). BMI did not significantly influence the frequency of staple-line bleeds overall (interaction *p* value for BMI, *p* = 0.4008). Staple-line bleeding was of a lower severity in the PSD-V group than the control (Table 5, *p* = 0.0002). Examination of post-operative bleed rates alone showed no statistically significant difference between the PSD-V and control groups (0/51 [0 %] vs 2/49 [4.1 %] patients; *p* = 0.2376).

**Table 3:** Staple-line bleeds and leaks during sleeve gastrectomy surgery and in the subsequent 30 days for the full population

	PSD-V		Control			
	% (n/N)	95 % exact CI	% (n/N)	95 % exact CI	Difference (95 % exact CI)	*p* value^a^
Primary objective						
Staple-line bleeds^b^	45.1 (23/51)	31.1, 59.7	79.6 (39/49)	65.7, 89.8	−34.5 % (−52.2, −15.7)	0.0005
Staple-line leaks^c^	0.0 (0/51)	0.0, 7.0	0.0 (0/49)	0.0, 7.3		

*CI* confidence interval, *PSD*-*V* Peri-Strips Dry® with Veritas®

^a^Fisher’s exact test used to compare categorical measures between groups

^b^Patients with any reported bleed from the intra-operative visit through day 30

^c^Patients with any reported leak the intra-operative visit through day 30

**Table 4:** Staple-line bleeds and leaks during sleeve gastrectomy surgery and in the subsequent 30 days according to BMI

	PSD-V, % (n/N)	Control, % (n/N)	Difference (95 % exact CI)	*p* value^a^	Interaction *p* value
Staple-line bleeds based on BMI^b^					
BMI <43	33.3 (7/21)	79.3 (23/29)	−46.0 (−68.4, −18.3)	0.0015	0.4008
BMI ≥43	53.3 (16/30)	80.0 (16/20)	−26.7 (−52.7, 2.4)	0.0742	

*BMI* body mass index, *CI* confidence interval, *PSD*-*V* Peri-Strips Dry® with Veritas®

^a^Fisher’s exact test used to compare between groups

^b^Patients with any reported bleed from the intra-operative visit through day 30 (no leaks were reported)

**Table 5:** Severity of staple-line bleeds during sleeve gastrectomy and in the subsequent 30 days

Severity of worst bleed	% (n/N)	
	PSD-V^a^, *N* = 51	Control, *N* = 49
No bleed	54.9 (28/51)	20.4 (10/49)
Mild bleed	43.1 (22/51)	67.4 (33/49)
Moderate bleed	2.0 (1/51)	8.2 (4/49)
Severe bleed	0.0 (0/51)	4.1 (2/49)

*PSD*-*V* Peri-Strips Dry® with Veritas®

^a^Wilcoxon rank-sum test used to assess the trend in severity of bleeds between the groups (*p* = 0.0002)

In cases where the 36 Fr bougie was used, significantly fewer patients in the PSD-V group experienced bleeds compared with patients in the control group (13/28 [46.4 %] vs 31/34 [91.2 %] patients; *p* = 0.0002). In contrast, in patients treated with a 34 Fr bougie, there was no significant difference between the treatment arms in the proportion of patients with staple-line bleeds (PSD-V group, 10/23 [43.5 %] patients, control group, 8/15 [53.3 %]; *p* = 0.7409). A significant difference in treatment effect was observed across bougie sizes (*p* = 0.03).

The average surgical time was statistically significantly shorter in the PSD-V group compared with the control group (58.8 vs 72.8 min; *p* = 0.0153 [Table 6]), and fewer patients required hemostatic clips and/or surgical sutures (10/51 [19.6 %] vs 33/49 [67.3 %] patients, *p* < 0.0001). Eight patients required placement of multiple sutures along the staple line (PSD-V group, *n* = 2; control group, *n* = 6). Two patients in the control group were returned to the operating room on post-operative day 1 due to bleeding.

**Table 6:** Intra-operative data summary

		PSD-V, *N* = 51	Control, *N* = 49	*p* value^a^
Surgery time (min)	Mean ± SD	58.8 ± 19.7	72.8 ± 25.8	0.0153
	Median (IQR)	55.0 (45.0, 70.0)	67.0 (48.0, 90.0)	
	Range	20.0–110.0	35.0–130.0	
Size of bougie				0.1538
34 Fr	% (n/N)	45.1 (23/51)	30.6 (15/49)	N/A
36 Fr	% (n/N)	54.9 (28/51)	69.4 (34/49)	N/A
Drain placed during surgery	% (n/N)	88.2 (45/51)	91.8 (45/49)	0.7412

*Fr* French bougie, *IQR* interquartile range, *NA* not applicable, *PSD*-*V* Peri-Strips Dry® with Veritas®, *SD* standard deviation

^a^Wilcoxon rank-sum test used to compare continuous measures between groups; Fisher’s exact test was used to compare categorical measures

There was no mortality in either treatment arm. In the PSD-V group, two (3.9 %) patients experienced AEs (gastroenteritis, *n* = 1; intra-abdominal abscess [retrocecal psoas abscess], *n* = 1). Five (10.2 %) patients in the control group experienced AEs: vomiting, *n* = 2; gastroenteritis, *n* = 1, and severe hemorrhage, *n* = 2. Both patients with severe hemorrhage were returned to the operating room on post-operative day 1 for corrective surgery.

## Discussion

In this single-center, randomized study, significantly fewer staple-line bleeds were observed between the intra-operative visit and day 30 with the use of PSD-V in comparison with the control group receiving no staple-line reinforcement. The staple-line bleeds in the PSD-V group were also of a lower severity. These results are consistent with reports of a reduced rate of staple-line failures with the use of bovine pericardium as buttress material in sleeve gastrectomy [13, 19].

BMI did not significantly influence the frequency of staple-line bleeds overall. However, subgroup analyses according to BMI demonstrated fewer staple-line bleeds in patients with a BMI ≤43 and in patients who received PSD-V compared with the control group, but no difference was observed between treatment groups in patients with a BMI >43. Tissue thickness may be an important factor to consider in interpreting these results, as a recent study examining the thickness of excised sleeve gastrectomy specimens demonstrated that male gender and BMI >50 kg/m^2^ were associated with thicker tissue in the antrum [23]. This is of particular relevance to the present study, as more males than females were recruited. To our knowledge, the influence of BMI in studies of staple-line reinforcement has not previously been examined; therefore, further investigations are warranted to confirm the results of the present study.

Bougie size was also found to significantly influence the frequency of staple-line bleeds, with fewer bleeds overall in the group that received the 34 Fr bougie compared with the 36 Fr bougie. However, the use of PSD-V was associated with a lower bleed rate compared with the control group in patients who received the 36 Fr bougie, whereas no difference was observed between treatment groups in patients who received the 34 Fr bougie. The reasons for this are unclear, and further studies are needed to clarify these results.

No staple-line leaks were observed in either treatment group, and therefore this study does not allow for inferences regarding efficacy in gastric leaks. However, Stamou et al., 2011 demonstrated no significant difference in staple-line leak rates between patients who received PSD-V in sleeve gastrectomy versus those who did not [14]. A review of the literature by Chen et al. questioned the benefits of staple-line reinforcement in reducing leaks, and this may be an area for clarification in the future.[25]

Surgical time was significantly reduced in the PSD-V group compared with the control group, with a difference of 14 min equating to almost 25 % of the PSD-V procedure time. The use of hemostatic clips and/or surgical sutures was also significantly reduced with the use of PSD-V. A limitation of the study is that the time for induction and reversal of anesthesia was excluded in order to demonstrate real surgical time differences for sleeve gastrectomy, and this may be an area for future investigation. Intra-operative time can be extended by bleeding complications, and the need for suturing and clipping. This is of considerable importance, as patients who require sleeve gastrectomy frequently have comorbidities in addition to morbid obesity, putting them at higher surgical risk. Any factor that can reduce intraoperative bleeding reduces the time that high-risk patients are kept under anesthesia, in turn reducing the risk of additional surgical complications.

A further limitation to the present study is that it was conducted at a single center, with a single surgical team, and therefore the sample size is small. Further studies with larger samples sizes would be beneficial to provide support for the results.

There were no deaths during the study, and there was a low incidence of AEs across the treatment groups. No safety issues were raised with the use of PSD-V, in line with other studies demonstrating that buttressing with bovine pericardium is readily accomplished and well tolerated [19]. Stamou et al. also reported that the use of PSD-V was associated with fewer days of hospitalization for patients who had undergone sleeve gastrectomy [14]. Thus, although the use of reinforcement may incur additional costs, it is possible that this may be offset by reductions in the number of days of hospitalization and is an important consideration for future studies.

## Conclusion

In conclusion, the use of PSD-V in patients with obesity alone, and those who also had a history of hypertension, diabetes, and/or sleep apnea, resulted in statistically significant decreases in the incidence and severity of intra-operative staple-line bleeding and surgical time. Our results suggest that PSD-V is a viable option for staple-line reinforcement in patients undergoing sleeve gastrectomy.

## References

[1] World Health Organization. Obesity and overweight; factsheet number 311. 2013. [Cited March 2014.] Available from: http://www.who.int/mediacentre/factsheets/fs311/en/.

[2] World Health Organization. Obesity and overweight. [Cited January 2014.] Available from: http://www.who.int/dietphysicalactivity/media/en/gsfs_obesity.pdf.

[3] Ministry of Health and Family Welfare Government of India. National family health survey 2005–2006 (NFHS-3). 2007. [Cited March 2014.] Available from: http://www.measuredhs.com/pubs/pdf/FRIND3/FRIND3-Vol1AndVol2.pdf.

[4] Deurenberg-Yap M, Chew SK, Lin VF, Tan BY, van Staveren WA, Deurenberg P (2001). Relationships between indices of obesity and its co-morbidities in multi-ethnic Singapore. Int J Obes Relat Metab Disord.

[5] Vikram NK, Pandey RM, Misra A, Sharma R, Rama Devi J, Khanna N (2003). Non-obese (body mass index < 25 kg/m2) Asian Indians with normal waist circumference have high cardiovascular risk. Nutrition.

[6] Misra A, Chowbey P, Makkar BM (2009). Consensus statement for diagnosis of obesity, abdominal obesity and the metabolic syndrome for Asian Indians and recommendations for physical activity, medical and surgical management. J Assoc Physicians India.

[7] Mechanick JI, Youdim A, Jones DB (2013). Clinical practice guidelines for the perioperative nutritional, metabolic, and nonsurgical support of the bariatric surgery patient—2013 update: cosponsored by American Association of Clinical Endocrinologists, The Obesity Society, and American Society for Metabolic & Bariatric Surgery. Obesity (Silver Spring).

[8] Silecchia G, Boru C, Pecchia A (2006). Effectiveness of laparoscopic sleeve gastrectomy (first stage of biliopancreatic diversion with duodenal switch) on co-morbidities in super-obese high-risk patients. Obes Surg.

[9] Sajid MS, Khatri K, Singh K, Sayegh M (2011). Use of staple-line reinforcement in laparoscopic gastric bypass surgery: a meta-analysis. Surg Endosc.

[10] Finks JF, Carlin A, Share D (2011). Effect of surgical techniques on clinical outcomes after laparoscopic gastric bypass—results from the Michigan Bariatric Surgery Collaborative. Surg Obes Relat Dis.

[11] Pinheiro JS, Correa JL, Cohen RV, Novaes JA, Schiavon CA (2006). Staple line reinforcement with new biomaterial increased burst strength pressure: an animal study. Surg Obes Relat Dis.

[12] Kawamura M, Kase K, Sawafuji M, Watanabe M, Horinouchi H, Kobayashi K (2001). Staple-line reinforcement with a new type of polyglycolic acid felt. Surg Laparosc Endosc Percutan Tech.

[13] Shikora S (2004). The use of staple-line reinforcement during laparoscopic gastric bypass. Obes Surg.

[14] Stamou K, Menenakos E, Dardamanis D (2011). Prospective comparative study of the efficacy of staple-line reinforcement in laparoscopic sleeve gastrectomy. Surg Endosc.

[15] Alley J, Fenton S, Harnisch M, Angeletti M, Peterson R (2011). Integrated bioabsorbable tissue reinforcement in laparoscopic sleeve gastrectomy. Obes Surg.

[16] Nguyen NT, Longoria M, Welbourne S, Sabio A, Wilson SE (2005). Glycolide copolymer staple-line reinforcement reduces staple site bleeding during laparoscopic gastric bypass: a prospective randomized trial. Arch Surg.

[17] Dapri G, Cadière G, Himpens J (2010). Reinforcing the staple line during laparoscopic sleeve gastrectomy: prospective randomized clinical study comparing three different techniques. Obes Surg.

[18] Albanopoulos K, Alevizos L, Flessas J (2012). Reinforcing the staple line during laparoscopic sleeve gastrectomy: prospective randomized clinical study comparing two different techniques. Preliminary results. Obes Surg.

[19] Hajj G, Haddad J (2013). Preventing staple-line leak in sleeve gastrectomy: reinforcement with bovine pericardium vs oversewing. Obes Surg.

[20] Indian Council of Medical Research. Ethical guidelines for biomedical research on human participants. 2006.[Cited March 2014]. Available from: http://icmr.nic.in/ethical_guidelines.pdf.

[21] World Medical Association. WMA Declaration of Helsinki—ethical principles for medical research involving human subjects. 2008. [Cited March 2014.] Available from: http://www.wma.net/en/30publications/10policies/b3/index.html.

[22] Rosenthal RJ, Diaz AA, Arvidsson D (2012). International Sleeve Gastrectomy Expert Panel Consensus Statement: best practice guidelines based on experience of >12,000 cases. Surg Obes Relat Dis.

[23] Rawlins L, Rawlins MP, Teel D (2014). Human tissue thickness measurements from excised sleeve gastrectomy specimens. Surg Endoc.

[24] Baker RS, Foote J, Kemmeter P, Brady R, Vroegop T, Serveld M (2004). The science of stapling and leaks. Obes Surg.

[25] Chen B, Kiriakopoulos A, Tsakayannis D, Wachtel M, Linos D, Frezza E (2009). Reinforcement does not necessarily reduce the rate of staple line leaks after sleeve gastrectomy. A review of the literature and clinical experiences. Obes Surg.

